# European Organization for Research and Treatment of Cancer Quality of Life Questionnaire Core 30: factorial models to Brazilian cancer patients

**DOI:** 10.1590/S1679-45082018AO4132

**Published:** 2018-04-19

**Authors:** Juliana Alvares Duarte Bonini Campos, Maria Cláudia Bernardes Spexoto, Wanderson Roberto da Silva, Sergio Vicente Serrano, João Marôco

**Affiliations:** 1 Universidade Estadual Paulista “Júlio de Mesquita Filho”, Araraquara, SP, Brazil.; 2 Hospital de Câncer de Barretos, Barretos, SP, Brazil.; 3 Instituto Universitário de Ciências Psicológicas, Sociais e da Vida, Lisboa, PT, Portugal.

**Keywords:** Quality of life, Neoplasms, Validation studies, Psychometrics, Qualidade de vida, Neoplasias, Estudos de validação, Psicometria

## Abstract

**Objective:**

To evaluate the psychometric properties of the seven theoretical models proposed in the literature for European Organization for Research and Treatment of Cancer Quality of Life Questionnaire Core 30 (EORTC QLQ-C30), when applied to a sample of Brazilian cancer patients.

**Methods:**

Content and construct validity (factorial, convergent, discriminant) were estimated. Confirmatory factor analysis was performed. Convergent validity was analyzed using the average variance extracted. Discriminant validity was analyzed using correlational analysis. Internal consistency and composite reliability were used to assess the reliability of instrument.

**Results:**

A total of 1,020 cancer patients participated. The mean age was 53.3±13.0 years, and 62% were female. All models showed adequate factorial validity for the study sample. Convergent and discriminant validities and the reliability were compromised in all of the models for all of the single items referring to symptoms, as well as for the “physical function” and “cognitive function” factors.

**Conclusion:**

All theoretical models assessed in this study presented adequate factorial validity when applied to Brazilian cancer patients. The choice of the best model for use in research and/or clinical protocols should be centered on the purpose and underlying theory of each model.

## RESUMO

**Objetivo:**

Avaliar as propriedades psicométricas dos sete modelos teóricos propostos na literatura para o *European Organization for Research and Treatment of Cancer Quality of Life Questionnaire Core 30* (EORTC QLQ-C30), quando aplicados em uma amostra de pacientes brasileiros com câncer.

**Métodos:**

As validades de conteúdo e de construto (fatorial, convergente, discriminante) foram avaliadas. Foi realizada análise fatorial confirmatória. A validade convergente foi avaliada pela variância média extraída. A validade discriminante foi analisada por meio de análise correlacional. A consistência interna e a confiabilidade composta foram utilizadas para avaliar a confiabilidade do instrumento.

**Resultados:**

Participaram do estudo 1.020 pacientes com câncer. A média de idade foi 53,3±13,0 anos, e 62% eram do sexo feminino. Todos os modelos mostraram validade fatorial adequada para a amostra de estudo. As validades convergente e discriminante e a confiabilidade foram comprometidas em todos os modelos para todos os itens isolados referentes aos sintomas, bem como para os fatores “função física” e “função cognitiva”.

**Conclusão:**

Todos os modelos teóricos avaliados neste estudo apresentaram validade fatorial adequada para pacientes brasileiros com câncer. A escolha do melhor modelo para uso em pesquisa e/ou protocolos clínicos deve ser baseada na finalidade e na teoria subjacente de cada modelo.

## INTRODUCTION

As diagnostic techniques and treatments evolve and become more modern in the field of oncology, patients have longer survival. Therefore, it is necessary to understand patients' quality of life.^(^ ^1^ ^,^ ^2^ ^)^ In clinical practice, the results of this investigation can be relevant to guide physicians and interdisciplinary teams in decisionmaking, by implementing treatment protocols.

Estimating quality of life is a complex task. It can be measured using several dimensions, such as patient's health, functional capacity, symptoms, psychosocial well-being, and life satisfaction.^(^ ^3^ ^)^ In an attempt to minimize bias and/or difficulty in interpreting results of this estimate, different theories and instruments have been developed to address the concept of quality of life. Each focuses on different aspects inherent to this construct.

Specifically for cancer patients, some studies focused on aspects related to health, disease and treatment.^(^ ^3^ ^,^ ^4^ ^)^ One of the instruments most often used in this investigation is the European Organization for Research and Treatment of Cancer Quality of Life Questionnaire Core 30 (EORTC QLQ-C30), created as an initiative of the Quality of Life Group, from the EORTC.^(^ ^5^ ^)^ The EORTC QLQ-C30 is currently on its third version. It was originally developed in Belgium, and it is currently available in many country-specific versions, including United States,^(^ ^6^ ^)^ Turkey,^(^ ^7^ ^)^ Germany,^(^ ^8^ ^)^ Singapore,^(^ ^9^ ^)^ Thailand,^(^ ^10^ ^)^ Spain,^(^ ^11^ ^)^ China,^(^ ^12^ ^)^ Indonesia,^(^ ^13^ ^)^ Mexico,^(^ ^14^ ^)^ Lebanon,^(^ ^15^ ^)^ Brazil^(^ ^16^ ^)^ and Morocco.^(^ ^17^ ^)^

The instrument is composed of 30 items, divided into five functioning scales (physical, role, cognitive, emotional, and social), three symptom scales (fatigue, pain, nausea and vomiting), one scale that evaluates overall quality of life, five single terms (dyspnea, sleep disturbance, appetite loss, constipation, and diarrhea), and one separate item to evaluate financial impact.^(^ ^5^ ^)^ Responses are given on a 4-point Likert scale, except for the items that evaluate overall quality of life (items 29 and 30), which are given on a 7-point Likert scale.

In the literature, there are currently seven proposals for theoretical models that can be used to interpret the EORTC QLQ-C30. These proposals were tested by Gundy et al.,^(^ ^18^ ^)^ on a sample obtained from a database including 48 countries. The first proposal is based on a standard model composed of nine first-order factors and five single terms, grouped together into a factor referred to as “spurious”. The second and third proposals are aligned with models composed of nine first-order factors and two second-order factors (“physical health” and “mental health”). The fourth proposal also has nine first-order factors and two second-order factors; however, the second-order factors in this proposal are referred to as “burden symptom” and “function”. In the fifth proposal, these two second-order factors are combined into a single factor known as “health-related quality of life”. These five models are reflective and oblique. The last two proposals are formative (causal) models, one with and one without path analysis. The authors concluded that all proposals had adequate validity for the sample; however, they pointed out the formative models were slightly inferior to that of other models.

Despite the extensive use of the EORTC QLQ-C30 in studies around the world, and the existence of theoretical model proposals for the questionnaire, no studies on Brazilian samples that assessed metric properties of the questionnaire were found in the literature, nor were any studies that tested all seven theoretical models presented in the literature.

## OBJECTIVE

To assess the psychometric properties of the theoretical models proposed in the literature for the EORTC QLQ-C30 when applied to a sample of Brazilian cancer patients.

## METHODS

### Sampling and study design

A cross-sectional study with a non-probabilistic sampling design was used. In this study, a total of 1,099 cancer patients receiving treatment at the *Hospital de Câncer de Barretos*, in the State of São Paulo, Brazil, were invited to participate.

The minimum sample size was calculated based on the recommendations by Hair Jr. et al.,^(^ ^19^ ^)^ who suggested the need for five to ten subjects for each model parameter. In addition, in the theoretical proposals of the EORTC QLQ-C30 presented by Gundy et al.,^(^ ^18^ ^)^ the maximum number of parameters was 41. Therefore, the minimum sample size was between 205 and 410 individuals. Since stability of the instrument in independent samples was also tested in the current study, the use of a second sample of the same size was necessary. In light of the lack of Brazilian studies assessing the psychometric qualities of the EORTC QLQ-C30 on Brazilian cancer patient populations, we decided to use a large enough sample to conveniently capture population variability.^(^ ^20^ ^)^

The following exclusion criteria were adopted: patients who had undergone medium- or large-scale surgeries, patients with cognitive deficits, patients receiving palliative care, patients with severe psychiatric disorders, and patients aged under 18 years.

To characterize the sample, clinical and demographic data on the patients were collected. The demographic characteristics included sex, age, socioeconomic class, and schooling level of the head of household. Age was measured as years completed, and socioeconomic class and schooling levels were classified according to the Brazilian Economic Classification Criteria.^(^ ^21^ ^)^ The clinical data considered at the outpatients clinic were presence of a defined diagnosis (yes/no), time interval since diagnosis (in months), type of cancer (classified by area and based on tumor location), staging of the disease (radiation therapy, chemotherapy associated with radiation therapy, hormone therapy, or immunotherapy), and the presence of metastasis (yes/no).

It is important to mention that not all patients answered all questions of the demographic inventory, and some clinical data were not found in some medical history reports.

### Measurement instrument

The impact of cancer and its treatment on quality of life was estimated using the third version of the EORTC QLQ-C30, which was proposed by the EORTC.^(^ ^5^ ^)^ The original Portuguese version of EORTC QLQ-C30 presented by the EORTC was used for data collection.

### Data collection

Patients were recruited in the waiting rooms of the outpatient's clinic (97.0%) and in the clinical inpatients unit of the hospital (3.0%). The data were collected from 2013 to 2014 by means of personal interviews, at the outpatient's clinic of the *Hospital de Câncer de Barretos*, by an interviewer who had been properly trained in a pilot study.

### Psychometric properties

The current validity of the construct of EORTC QLQ-C30 was analyzed using the factorial, convergent, and discriminant validity.

### Factorial validity

In this study seven models (model a – Ma, model b – Mb, model c – Mc, model d – Md, model e – Me, model f – Mf, and model g – Mg) proposed for the EORTC QLQ-C30^(^ ^18^ ^)^ were assessed (Table 1). All models were tested for the study sample by confirmatory factor analysis (CFA), using the matrix of polychoric correlations and the estimation method weighed least squares mean and variance adjusted (WLSMV) implemented through MPLUS 7.2 software (Muthén & Muthén, Los Angeles, CA, USA). The χ^2^ ratio and degrees of freedom (df) were used as indexes to assess the quality of fit (χ^2^/df). In addition, the comparative fit index (CFI), goodness of fit index (GFI), Tucker Lewis index (TLI) and the root mean square error of approximation (RMSEA) with 90% confidence interval were also used.^(^ ^22^ ^)^ The fit of the model was considered adequate when χ^2^/df ≤2.0, CFI and TLI ≥0.90, and RMSEA ≤0.10.^(^ ^22^ ^)^ Root mean square error of approximation values were used to compare the models; those with lower values were chosen as the best one.^(^ ^23^ ^)^

**Table 1:** Complete models evaluated of the European Organization for Research and Treatment of Cancer Quality of Life Questionnaire Core 30 (EORTC QLQ-C30)

Model	Second-order factor	First-order factor	Items	Correlated factors
a	-	Quality of life	→29, 30	All
	-	Physical function	→1, 2, 3, 4, 5	
	-	Role function	→6, 7	
	-	Emotional function	→21, 22, 23, 24	
	-	Cognitive function	→20, 25	
	-	Social function	→26, 27	
	-	Fatigue	→10, 12, 18	
	-	Nausea and vomiting	→14, 15	
	-	Pain	→9, 19	
	-	Spurious	→8, 11, 13, 16, 17	
b	-	Quality of life	→29, 30	Quality of life, mental health and physical health
	Physical health	→Physical function	→1, 2, 3, 4, 5	
		→Nausea and vomiting	→14, 15	
		-	→11, 13, 16, 17	
	Mental health	→Emotional function	→21, 22, 23, 24	
		→Cognitive function	→20, 25	
	Both factors (mental health and physical health)	→Social function	→26, 27	
		→Fatigue	→10, 12, 18	
		→Role function	→6, 7	
		→Pain	→9, 19	
		-	→8	
b adapted	-	Quality of life	→29, 30	Quality of life, mental health and physical health
	Physical health	→Physical function	→1, 2, 3, 4, 5	
		→Nausea and vomiting	→14, 15	
		→Role function	→6, 7	
		→Fatigue	→10, 12, 18	
		→Pain	→9, 19	
		-	→8, 11, 13, 16, 17	
	Mental health	→Emotional function	→21, 22, 23, 24	
		→Cognitive function	→20, 25	
		→Social function	→26, 27	
		→Pain	→9, 19	
		-	→8	
c	-	Quality of life	→29, 30	Quality of life, mental function and physical burden
	Physical burden	→Physical function	→1, 2, 3, 4, 5	
		→Fatigue	→10, 12, 18	
		→Nausea and vomiting	→14, 15	
		→Pain	→9, 19	
		-	→8, 11, 13, 16, 17	
	Mental function	→Emotional function	→21, 22, 23, 24	
		→Cognitive function	→20, 25	
	Both factors (mental function and physical burden)	→Role function	→6, 7	
		→Social function	→26, 27	
c adapted	-	Quality of life	→29, 30	Quality of life, mental function and physical burden
	Physical burden	→Physical function	→1, 2, 3, 4, 5	
		→Fatigue	→10, 12, 18	
		→Nausea and vomiting	→14, 15	
		→Pain	→9,19	
		-	→8, 11, 13, 16, 17	
		→Role function	→6, 7	
	Mental function	→Emotional function	→21, 22, 23, 24	
		→Cognitive function	→20, 25	
		→Social function	→26, 27	
d	-	Quality of life	→29, 30	Quality of life, function and burden
	Function	→Physical function	→1, 2, 3, 4, 5	
		→Role function	→6, 7	
		→Emotional function	→21, 22, 23, 24	
		→Cognitive function	→20, 25	
		→Social function	→26, 27	
	Burden	→Fatigue	→10, 12, 18	
		→Nausea and Vomiting	→14, 15	
		→Pain	→9, 19	
		-	→8, 11, 13, 16, 17	
e	-	Quality of life	→29, 30	Quality of life and health-related quality of life
	Health-related quality of life	→Physical function	→1, 2, 3, 4, 5	
		→Role function	→6, 7	
		→Emotional function	→21, 22, 23, 24	
		→Cognitive function	→20, 25	
		→Social function	→26, 27	
		→Fatigue	→10, 12, 18	
		→Nausea and Vomiting	→14, 15	
		→Pain	→9, 19	
		-	→8, 11, 13, 16, 17	
f* and g†		Quality of life	→29, 30	None
	Function	→Physical function	→1, 2, 3, 4, 5	
		→Role function	→6, 7	
		→Emotional function	→21, 22, 23, 24	
		→Cognitive function	→20, 25	
		→Social function	→26, 27	
	Burden	←Fatigue	→10, 12, 18	
		←Nausea and vomiting	→14, 15	
		←Pain	→9, 19	
		-	←8, 11, 13, 16, 17	
		→Quality of life and function	-	

* formative first-order factors with freely estimated weights;

† formative first-order factors with fixed weights. The arrows refer to the direction of the trajectories that must be used to build the model.

The items that presented factor weights (λ) <0.40 were removed from the models, like the items found to be redundant by the modification indices estimated by Lagrange multiplier method (LM >11; p< 0.001). Modification indices were also used to check for correlations between item errors.^(^ ^22^ ^)^

### Convergent validity

To assess the convergent validity of the EORTC QLQ-C30, the average extracted variance (AVE) was calculated.^(^ ^24^ ^)^ Values of AVE ≥0.50 were considered adequate.^(^ ^22^ ^)^

### Discriminant validity

Discriminant validity was estimated using correlational analysis between the factors, and values were considered adequate when AVE and AVE ≥r_ij_^2^ (square of the correlation between the factors).^(^ ^22^ ^-^ ^24^ ^)^

### Reliability

Reliability was estimated using the composite reliability (CR)^(^ ^24^ ^)^ and Cronbach's α coefficient.^(^ ^25^ ^)^ Values of CR and α ≥0.70 were considered adequate.

### Ethical considerations

This study was approved by the Ethics Committee for Human Research at the *Hospital de Câncer de Barretos* (under no. 561/2011). Only patients who agreed to and signed the Informed Consent Form were included in the study.

## RESULTS

A total of 1,020 patients (compliance rate of 92.8%) with defined neoplasia diagnosis participated. The majority were women (n=633), were interviewed in the ambulatory center (96.9%), and belonged to the socioeconomic class C (estimated monthly household income: R$1.147,00 to R$1.685,00. The average age of the participants was 53.3±13.0 years. As for the type of neoplasia, 31.2% of patients had breast cancer; 20.0% cancer of the lower digestive tract; 11.1% gynecological cancers; 9.6% cancer of the upper digestive tract; 7.4% cancers of the head and neck; 7.4% urological cancers; and 13.3% had other types of cancer. Most patients were in stage III (38.5%) of the disease, and their cancers had not metastasized (61.8%). The number of patients in stages I and II totaled 34.0%. Chemotherapy was the most prevalent treatment (64.0%), followed by radiation therapy (17%) and chemotherapy associated with radiation therapy (15%).

The information on the confirmatory factor analysis of the different models of the EORTC QLQ-C30 can be found in table 2. It should be clarified that when the proposed EORTC QLQ-C30 factorial structures were analyzed, the Mb and Mc, presented by Gundy et al.,^(^ ^18^ ^)^ did not fit the sample. In order for them to be fitted it was necessary to remove some shared trajectories so that the models were modified. These models were called “adapted”. This adaptation was made by the researchers based on the theoretical approximation of the content of the first order factors and/or items with the second order factors.

**Table 2:** Indicators for evaluation of psychometric properties of factor models of the European Organization for Research and Treatment of Cancer Quality of Life Questionnaire Core 30 (EORTC QLQ-C30)

Model	CFA							e	AVE	CR	α
	λ	β (p<0.001)	χ^2^/df	CFI	TLI	RMSEA (90%CI)	r^2^				
a complete*	0.22-1.00	-	3.07	0.97	0.96	0.045 (0.042-0.049)	0.05-0.83	-	0.21-0.88	0.54-0.94	0.40-0.84
a refined†	0.45-1.00	-	3.59	0.97	0.96	0.051 (0.047-0.054)	0.05-0.83	16, 17	0.30-0.88	0.56-0.94	0.39-0.84
a refined† (without spurious factor)	0.53-1.00	-	3.99	0.97	0.96	0.055 (0.051-0.058)	0.05-0.83	-	0.55-0.88	0.66-0.94	0.40-0.84
b adapted	0.23-1.00	0.39-0.96	5.76	0.92	0.91	0.069 (0.066-0.072)	0.29-0.63	-	0.23-0.88	0.57-0.94	0.40-0.84
b adapted‡ refined†	0.48-1.00	0.39-0.97	6.60	0.92	0.91	0.075 (0.072-0.078)	0.29-0.63	16, 17	0.32-0.88	0.58-0.94	0.40-0.84
c adapted‡	0.22-1.00	0.59-0.95	3.54	0.96	0.95	0.050 (0.047-0.053)	0.32-0.65	-	0.25-0.88	0.59-0.94	0.40-0.84
c adapted‡ refined†	0.47-1.00	0.59-0.95	4.01	0.95	0.95	0.055 (0.052-0.058)	0.32-0.64	16, 17	0.36-0.88	0.62-0.93	0.30-0.84
d	0.23-0.99	0.57-0.98	3.99	0.95	0.95	0.055 (0.052-0.058)	0.30-0.83	-	0.27-0.87	0.62-0.93	0.40-0.84
d refined†	0.48-0.99	0.58-0.99	4.54	0.95	0.94	0.059 (0.056-0.062)	0.29-0.83	16, 17	0.39-0.87	0.65-0.93	0.30-0.84
e	0.22-0.99	0.57-0.94	3.99	0.95	0.94	0.055 (0.052-0.057)	0.38	-	0.24-0.87	0.58-0.93	0.40-0.84
e refined‡	0.47-1.00	0.57-0.94	4.53	0.95	0.94	0.059 (0.056-0.062)	0.38	16, 17	0.35-0.88	0.61-0.94	0.30-0.84
f	0.54-1.00	0.20-1.00	3.73	0.96	0.95	0.052 (0.049-0.055)	-	-	0.18-0.88	0.49-0.88	0.40-0.84
g	0.54-1.00	0.20-1.00	3.74	0.96	0.95	0.052 (0.049-0.055)	-	-	0.19-0.88	0.51-0.93	0.40-0.84

* original;

† fitted;

‡ adapted model by authors.

CFA: confirmatory factor analysis: λ: factor weight; χ^2^/df: χ^2^ ratio by degree of freedom; CFI: comparative fit index; TLI: Tucker Lewis index; RMSEA: root mean square error of approximation; r^2^: square of the correlation between the factors; e: excluded items; AVE: average variance extracted; CR: composite reliability; α: Cronbach alpha coefficient.

For Ma, Mb, Mc, Md, and Me, items 16 and 17 presented inadequate factor weights and were excluded. The Mf and Mg showed adequate fit without changes. The seven factorial models tested, after refinement, presented adequate fit to the data. Comparatively, Mb presented higher RMSEA values. Convergent and discriminant validities have been compromised in all models for isolated items relating to symptoms and for the factors from physical and cognitive functions. The values found in the composite reliability and internal consistency showed that the models for the isolated items relating to symptoms and for the factor of cognitive function did not present adequate reliability.

## DISCUSSION

The objective of this study was to estimate, for very first time in the literature, the psychometric properties of the seven factorial models proposed for the EORTC QLQ-C30, when applied to a sample of Brazilian cancer patients. The results showed that all models proposed exhibited satisfactory factorial validity for the sample. These findings are consistent with those reported by Gundy et al.^(^ ^18^ ^)^ However, for the data presented by these authors, the Mb was considered the most indicated. In our study, although this model presented adequate fit to the data, comparing to the other models (*e.g.*, RMSEA values), this does not seem to be the model of choice. Given the adequacy of the models to the data, we suggest that the choice of the model to be used in clinical context and/or screening should be guided by the investigation objective. Thus, this choice will be centered in the underlying theory for the elaboration of each model, since that statistically all the proposals presented adequate factorial validity.

Among the limitations found in the psychometric properties of EORTC QLQ-C30 we can highlight the high χ^2^ values observed in the CFA, the low convergent and discriminant validity and reliability. The χ^2^ values were probably increased due to the high sensitivity of this index to the sample size^(^ ^26^ ^)^ and, therefore, this aspect does not characterize a limitation for the factorial validity of the models. Regarding to the low convergent and discriminant validity of the isolated items, this fact may have occurred due to the theoretical approach between the contents of the same and the other factors of the instrument. In contrast, the low reliability observed may be a reflection of the reduced number of items per factor, and the grouping of items with low factorial weights in the same factor. This aspect can not be considered a limitation of the EORTC QLQ-30, but only a warning signal when used in samples with similar characteristics to the present study, because it can not replicate in samples with different characteristics and/or contexts.

The items 16 and 17 had to be removed so that the reflective models presented adequate fit to the data (Ma, Mb, Mc, Md, Me). These items refer to gastrointestinal symptoms (constipation and diarrhea), and their inadequacy can be attributed to the characteristics of the study sample, *i.e.*, oncology outpatients. These symptoms in cancer patients may be less significant than in others, considering the impacts of disease and of treatment on quality of life.

One limitation of this study was the convenience sampling that resulted in data of cancer patients with specific clinical characteristics (*e.g.* outpatient care, with breast and lower digestive tract neoplasms, nonmetastatic, and on chemotherapy), making it difficult to generalize the evidence presented for the population of oncological patients. We attempted to minimize this limitation by using an extended sample size. However, we believe that despite this limitation, this study presented evidence of the validity and reliability of different theoretical models of EORTC QLQ-C30 for a Brazilian sample.

This evidence may be important to assess quality of life of cancer patients in clinical and epidemiological contexts, not only regarding the choice of the measurement instrument but also the theoretical model to be used and its operation.

## CONCLUSION

All theoretical models presented in the literature for the EORTC QLQ-C30 exhibit satisfactory factorial validity when applied to Brazilian cancer patients. Convergent and discriminant validity and reliability are useful for the cognitive function factor and for the single items related to symptoms.
